# The folded *k*-spectrum kernel: A machine learning approach to detecting transcription factor binding sites with gapped nucleotide dependencies

**DOI:** 10.1371/journal.pone.0185570

**Published:** 2017-10-05

**Authors:** Abdulkadir Elmas, Xiaodong Wang, Jacqueline M. Dresch

**Affiliations:** 1 Department of Electrical Engineering, Columbia University, New York, NY, United States of America; 2 Department of Mathematics and Computer Science, Clark University, Worcester, MA, United States of America; Harbin Institute of Technology Shenzhen Graduate School, CHINA

## Abstract

Understanding the molecular machinery involved in transcriptional regulation is central to improving our knowledge of an organism’s development, disease, and evolution. The building blocks of this complex molecular machinery are an organism’s genomic DNA sequence and transcription factor proteins. Despite the vast amount of sequence data now available for many model organisms, predicting where transcription factors bind, often referred to as ‘motif detection’ is still incredibly challenging. In this study, we develop a novel bioinformatic approach to binding site prediction. We do this by extending pre-existing SVM approaches in an unbiased way to include all possible gapped *k*-mers, representing different combinations of complex nucleotide dependencies within binding sites. We show the advantages of this new approach when compared to existing SVM approaches, through a rigorous set of cross-validation experiments. We also demonstrate the effectiveness of our new approach by reporting on its improved performance on a set of 127 genomic regions known to regulate gene expression along the anterio-posterior axis in early *Drosophila* embryos.

## Introduction

When studying the complex control of gene expression, often the first step is to locate an enhancer, or *cis*-regulatory module (CRM), within the genome. An enhancer is a non-coding region of DNA, typically located upstream of the promoter region, which binds transcription factor (TF) proteins and subsequently regulates the gene’s expression. This regulation is extremely important for many of the key processes involved in embryonic development [1, 2]. The discovery of clusters of key TF binding sites has been critical to identifying potential enhancers in the genome, and mapping out the organization of these binding sites has been crucial in guiding our understanding of enhancer function and evolution [2–4].

DNA motif discovery and sequence classification to identify portions of the genome with specific biological function(s) has been a central problem in computational biology, addressed by numerous approaches based on sequence alignment [5, 6], profiling consensus patterns of motifs [7, 8], and hidden Markov models [9, 10]. In this study, we draw from ideas introduced in Position Weight Matrix-based approaches to develop a novel, unbiased Support Vector Machine approach [11, 12].

### Position Weight Matrix approach

Since the 1980s, one of the most popular bioinformatic approaches for predicting transcription factor binding sites (TFBSs) involves constructing a Position Weight Matrix (PWM) and scanning a DNA sequence for subsequences with a high probability of binding the TF of interest [13–15]. Standard implementations of this approach rely on the assumption that nucleotide positions within a TFBS are independent of each other [16, 17]. Even with this simplifying assumption, these models have proven themselves effective in predicting binding sites in a variety of different species [16–20].

In an attempt to implement a more systematic and unbiased approach in predicting TFBSs, there have been numerous different extensions to this approach, relaxing the assumption of nucleotide independence of contiguous nucleotides within the binding site. Common extensions include dinucleotide and *k*-mer models, which allow for dependence between adjacent nucleotides or a contiguous string of *k* nucleotides, respectively [21–23]. Even more recently, an algorithm was developed, referred to as MARZ, that combinatorially considers all possible gapped nucleotide dependencies across a fixed number of nucleotides [11]. By considering all possible dependencies, this new algorithm includes traditional PWM models, dinucleotide and *k*-mer models, as well as models with noncontiguous (i.e. gapped) dependencies. When tested on a set of well characterized TFs in *Drosophila*, MARZ illustrated that gapped models often outperform traditional PWM-based models [11, 12]. Although this may not be the case for all TFs or in all species, these studies have highlighted the importance of using an unbiased approach and considering all possible combinations of nucleotide dependence when attempting to make robust predictions of TFBSs.

### Support Vector Machines

In recent years, discriminative approaches, such as Support Vector Machines (SVM), have been introduced and shown to be the best performing methods for sequence classification [24]. In the SVM classifier approach, the input sequences from different classes are considered as labeled examples and a learning algorithm is trained to find an optimal decision boundary between the different classes. The decision boundary (determined by a hyperplane in a multidimensional feature space) optimally separates the feature representations of the labeled examples. In this supervised approach, the unlabeled (test) sequences are later given to the algorithm, and the algorithm uses the learned decision boundary to predict labels/classes for these test sequences [25].

One of the earliest SVM approaches for biological sequence classification, which was originally implemented on protein sequences made up of strings of amino acids, was the Fisher kernel [26]. This approach, tested on the SCOP database [27], is based on a computationally-demanding generative model that requires one to build hidden Markov model profiles for each positive training sequence to obtain the feature vector representations. A new protein sequence is represented by a Fisher score vector, then its Kernel function for a given protein family is computed based on the Euclidean distance between this score vector and the score vectors that are precomputed for the known positive and negative examples of that protein family [26]. In a later study by [28], the SVM-pairwise kernel was introduced in which the pairwise alignments between each training sequence are used as the SVM features. This methodology is similar to the Fisher kernel approach and the process is computationally-expensive as well. In [29], a more efficient “spectrum” kernel was introduced for the classification of protein sequences, where the feature vector consists of the occurrences of all *k*-mer contiguous subsequences in a given amino acid sequence. In subsequent works, the authors extended their approach for “inexact” subsequence matches for the improved classification performance, which allows up to a certain number of mismatches when counting the *k*-mer occurrences [30, 31].

#### The advantage / extension of our approach

The *k*-mer spectrum is an effective representation of DNA sequences in terms of discriminating functional segments (i.e., TFBS, or promoter/enhancer regions) from the genomic background. The TFBSs can be characterized by the specific composition of the *k*-mer subsequences inherent to the binding preference of a TF protein, where the consensus motif sequence is defined as the most observed *k*-mer in the corresponding binding sites followed by those that differ from the consensus sequence in various degrees.

It has been suggested that protein binding sites have varying levels of nucleotide interdependencies, i.e., the nucleotide patterns in certain (non-adjacent) bases within binding sites may appear more often than the patterns in other bases [12, 32, 33]. This suggests a (gapped) dependency in some non-adjacent bases within the TFBSs which can be modeled by a “gapped” *k*-mer. The gapped *k*-mer is a length *k* sequence of nucleotides that breaks dependency in certain bases that cannot form a significant consensus, i.e., we can assume the interdependency between the first and the last nucleotides in the following 3 binding sites {“*AACA*”, “*ACGA*”, “*AGTA*”}, where a “gapped” *k*-mer represented by “*ANNA*” (where each ‘*N*’ represents a gap, corresponding to no specific nucleotide preference) will be a better fit to the majority of TFBSs (i.e., 3/3) than any other “contiguous” *k*-mer.

Following this intuition, to account for the TF proteins that possess nucleotide interdependency, a more suitable representation of the DNA binding sites –in terms of sequence discrimination– should incorporate the gapped *k*-mer compositions as well as the regular *k*-mers. In that respect, the composition (enrichment) of the specific gapped *k*-mers in the analyzed sequence (relative to the enrichments obtained from the training data) can help identify such TFs that possess interdependency in their preferred binding sites. In the following section, we describe a generalized discriminative approach for detecting functional DNA sequences including those that may possess nucleotide interdependency in particular TFBSs.

Although PWM-based approaches are often used for TFBS discovery, and have shown success in incorporating gapped dependencies, SVM approaches require much less prior knowledge of binding preferences [11, 12, 24, 25]. A PWM is built from a set of sequences known to bind a specific TF [11, 16, 17]. Thus, these studies focus on specific sets of TFs, often with the goal of identifying high affinity binding sites, and have no flexibility in discovering TFBSs for TFs not included in the initial input [11, 16, 17]. SVM approaches, on the other hand, require no knowledge of the TFs that may bind to the DNA search sequence or their binding preference within the genome; they are searching for enriched motifs within the search sequence with the goal of identifying TFBSs [24, 25]. Due to the extremely different inputs needed, and often-different underlying goals of using such algorithms, it is impossible to conduct a valid comparison of the results from PWM-based vs. SVM approaches.

### SVM for sequence classification

Support Vector Machine (SVM) is a discriminative learning method that finds an optimal decision boundary that separates, in the case of binary classification, the positive and negative data sets represented in a high-dimensional vector space [25]. Consider the training data set of labeled input vectors (***x***_*i*_, *y*_*i*_), *i* = 1, …, *t*, where $x_{i}\in R^{N}$ is the projection of the *i*-th input data into a high-dimensional feature space (obtained by a known mapping function, Ψ), and *y*_*i*_ ∈ {−1, 1} is the respective class label. In this classification problem, a basic (linear) decision boundary B can be found by minimizing ||***w***||^2^ subject to $y_{i}\left(x_{i}^{T}w+b\right)\geq 1$, *i* = 1, …, *t*, where $w\in R^{N}$ is the normal vector to the decision boundary (which is a hyperplane, i.e., $B=\{x\in R^{N}:x^{T}w+b=0\}$), *b* is the classifier bias, and the term $x_{i}^{T}w$ represents the inner product between the vectors ***x***_*i*_ and ***w***. For practical considerations a dual problem is rather solved to find the decision boundary by forming the Lagrangian (with multipliers *α*) for the above quadratic programming problem [24]:

$$\begin{array}{l} \text{max}\sum_{i}\alpha_{i}-\frac{1}{2}\sum_{i}\sum_{j}\alpha_{i}\alpha_{j}y_{i}y_{j}x_{i}^{T}x_{j},\\ \text{subject}\;\text{to}\;\alpha_{i}\geq 0\;\forall i, \end{array}$$ (1)

whereby the normal vector ***w*** can be optimally constructed from ***w*** = ∑_*i*_ *y*_*i*_*α*_*i*_***x***_*i*_. Here, the learned variables (***w***, b) serve as the parameters of the classification rule. A test example ***z*** is then classified by the expression

$$\begin{array}{c} f\left(z\right)=z^{T}w+b=\sum_{i}y_{i}\alpha_{i}z^{T}x_{i}+b, \end{array}$$

where *f*(***z***) represents the distance of ***z*** from the learned decision boundary, and its sign gives the estimated class label. The inner product in (1) yields a measure of similarity in the feature space F where the coordinates are defined by the vector elements. The similarity between any two input data (*χ*_*i*_, *χ*_*j*_) can be generalized by using the Kernel functions *K*(*χ*_*i*_, *χ*_*j*_) [24].

A convenient Kernel function for sequence classification is the *k-spectrum kernel*, [29] which describes the similarity of sequences by their (*k*-mer) subsequence compositions of a fixed length *k*. Consider that the training data *χ* is a character sequence belonging to an input space X consisting of all finite sequences from an alphabet A of size *ℓ*. For a given *k* ≥ 1, it is projected to a frequency vector called “*k-spectrum*”, i.e., ***x*** = Ψ_*k*_(*χ*), that represents the occurrences of all possible *k*-mer subsequences in *χ*

$$\begin{array}{c} \Psi_{k}\left(\chi \right)={\left(\phi_{\alpha }\left(\chi \right)\right)}_{\alpha \in A^{k}}, \end{array}$$

where *ϕ*_***α***_(*χ*) is the number of times ***α*** occurs in *χ*, and $A^{k}$ represents the set of all possible length-k sequences from the alphabet A.

In [29], the *k*-spectrum kernel’s mapping function Ψ_*k*_ considers only the subsequences of “contiguous” (dependent) nucleotides (i.e., *k*-mers). In a later related work, authors introduce the *mismatch kernel* to allow for a certain number of mismatches in the *k*-mer occurrences. Recently, a more systematic approach was proposed for detecting regulatory sequences possessing certain degrees of nucleotide interdependency through the search of sequence motifs called “gapped *k*-mers” [11]. In our study, we extend the concept of *k*-spectrum given in [29] by incorporating all types of nucleotide interdependency in *k*-mers to improve the predictive power of the feature set. That is, we extend the feature set of *k*-mers by those of the gapped *k*-mers given in [11] that ignore all possible subsets of nucleotides. As it will be explained next, the mapping function only evaluates the data points under the *k*-spectrum and computes the extended (gapped) features efficiently by folding this spectrum in certain coordinates specific to the gapped *k*-mer features.

Gapped *k*-mers have been recently used for regulatory sequence prediction in the SVM-based classifiers. In [34], the authors consider *ℓ*-mers with *k* non-gapped bases (*k* ≤ *ℓ*) and estimate *ℓk* feature frequencies based on the *ℓ*-mer mismatch profiles between sequences. Their model considers a fixed number of gaps, i.e., *ℓ* − *k*, when computing the SVM kernel. In contrast, we allow “variable-length” gaps (between 0 and *k* − 1) in the *k*-mer and thereby incorporate a full and unbiased list of sequence features in the SVM kernel. This also allows us to perform systematic analyses on the sequences which we describe next.

In the context of gapped dependency, another recent study [35] employed an SVM learning approach, repDNA, in which pseudo nucleotide compositions that represent the gapped dependency of nucleotides is incorporated into the *k*-mers, as well as more sophisticated features derived from the physicochemical properties of DNA.

### Folded *k*-spectrum kernel

We use the binary notation to refer the dependent nucleotides in a subseqeunce ***α***, where ‘1’ represents the dependent nucleotides and ‘0’ represents the gap. For example, given *k* = 3 (3-mer) the binary value of the decimal number 5 is 101; therefore 5 encodes the 3-mer sequences of which only the 1st and the 3rd nucleotides are interdependent. We denote this dependency model by a set of gapped *k*-mer features $A_{5}^{3}$ which consists of 16 sequences varying on the nucleotides (1,3), i.e., $A_{5}^{3}=\left\{ANA,ANC,ANG,ANT,CNA,\ldots ,TNG,TNT\right\}$, where the gaps are represented by ‘N’. Similarly, the decimal number 7 yields the binary sequence 111 and the corresponding $A_{7}^{3}$ represents the set of all (contiguous) 3-mer sequences. For *k* = 3, the remaining possible feature sets are $A_{3}^{3}$ and $A_{1}^{3}$ which consists of all possible dimer (011) and monomer (001) sequences, respectively.

As described above, there are 4 different feature sets (gapped *k*-mer models) for *k* = 3, i.e., monomer, dimer, 1-gapped, and 3-mer. In general, this number grows exponentially with *k*, i.e., 2^*k*−1^ different feature sets. However, in practice the length of *k* = 6 is a sufficient (and optimal) choice for the purpose of sequence discrimination [36, 37], which we also used in this study. In the following, we present a general formulation of the folded *k*-spectrum kernel for any *k*.

Using the same notation, we define the feature map of a particular feature set $A_{2m-1}^{k},m=1,\ldots ,2^{k-1}$ by

$$\begin{array}{c} \Phi_{\left(k,m\right)}\left(\chi \right)={\left(\phi_{\alpha }\left(\chi \right)\right)}_{\alpha \in A_{2m-1}^{k}}, \end{array}$$ (2)

where Φ_(*k*,*m*)_ maps the input data into the *m*-th feature set which can be denoted by $A_{2m-1}^{k}={\left(\left[\alpha \alpha \ldots \alpha \right]⊗{\left(2m-1\right)}_{\left(2\right)}\right)}_{\alpha \in A}$, with (*l*)_(2)_ representing the decimal *l* in binary sequence, i.e. (5)_(2)_ = 101. In Eq (2), we used the relative frequency mapping for *ϕ*_***α***_(*χ*) to cancel out the influence of different sequence lengths and different background frequencies of ***α***, i.e., it represents the number of times ***α*** occurs in *χ* divided by the number of possible *k*-mers (|*χ*| − *k* + 1), relative to the same measure observed in the genome

$$\begin{array}{c} \phi_{\alpha }\left(\chi \right)=\frac{\#\;\alpha \;occurs\;in\;\chi }{\left|\chi \right|-k+1}{\left(\frac{\#\;\alpha \;occurs\;in\;genome}{\left|genome\right|-k+1}\right)}^{-1}. \end{array}$$

Notice that in (2), a value of *m* = 2^*k*−1^ corresponds to the last feature set (which is the set of contiguous *k*-mers), where the term 2*m* − 1 yields the length-*k* sequence of 1s in the binary representation, i.e., (2*m* − 1)_(2)_ = (2 × 2^*k*−1^ − 1)_(2)_ = (2^*k*^ − 1)_(2)_ = {1}^*k*^ = [11…1]. Similary, *m* = 1 represents the monomer model with the feature set

$$A_{2m-1}^{k}=A_{1}^{k}=\left\{N\ldots NA,\;N\ldots NC,\;N\ldots NG,\;N\ldots NT\right\},$$

and *m* = 2 represents the dimer model with the feature set

$$\begin{array}{cc} A_{2m-1}^{k}=A_{3}^{k} & =\{N\ldots NAA,\;N\ldots NAC,\;N\ldots NAG,\\ & N\ldots NAT,\;N\ldots NCA,\;\ldots ,\;N\ldots NTT\}. \end{array}$$

Any other value between 2 < *m* < 2^*k*−1^ corresponds to the gapped feature sets given that *k* > 2. Considering all possible gapped *k*-mers, i.e., {*m* = 1, …, 2^*k*−1^}, the extended *k*-spectrum feature map is then given by

$$\begin{array}{c} \Psi_{k}\left(\chi \right)={\left(\Phi_{\left(k,m\right)}\left(\chi \right)\right)}_{m=1}^{2^{k-1}}\;. \end{array}$$ (3)

For efficiency, we only evaluate the contiguous *k*-mer features under the *k*-spectrum map, i.e., Φ_(*k*,2^*k*−1^)_(*χ*), then the mapping of *χ* to any other feature set (*m* < 2^*k*−1^) is calculated as a linear sum of the feature-specific values in this *k*-spectrum map, imitating a folding process over those coordinates. For example, given *k* = 3 the frequency of the feature sequence “*ANA*” in $A_{5}^{3}$, is computed by

$$\begin{array}{l} \phi_{ANA}\left(\chi \right)\;=\;\sum_{\alpha \in A}\phi_{A\alpha A}\left(\chi \right)\\ =\phi_{AAA}\left(\chi \right)+\phi_{ACA}\left(\chi \right)+\phi_{AGA}\left(\chi \right)+\phi_{ATA}\left(\chi \right), \end{array}$$

where [*ϕ*_*AAA*_(*χ*), *ϕ*_*ACA*_(*χ*), *ϕ*_*AGA*_(*χ*), *ϕ*_*ATA*_(*χ*)] are obtained from Φ_(*k*,2^*k*−1^)_(*χ*). In other words, the mapping function of a gapped *k*-mer feature (i.e., “*ANA*”) is the (unweighted) linear combination of the mapping functions of all contiguous *k*-mer features which differ from that gapped *k*-mer sequence in the gapped locations.

### Additional approaches

Many alternative approaches have been recently proposed in motif discovery and feature prediction. One such approach investigates the gapped nucleotide dependencies in terms of discriminating real pre-miRNA sequences from false sequences. In [38], authors use *k*-mer components to represent the RNA sequences, and they propose an approach (degenerate *k*-mer) to deal with the problem of over fitting. In another study, this method was modified and applied to DNA-binding proteins [35] based on building pseudo amino acid composition profiles and then incorporating gapped dependency between amino acid pairs through a reduced alphabet of amino acids. Features such as these pseudo components of DNA, RNA, or protein sequences are generated using previously published and available tools such as repDNA and Pse-in-One. [39, 40]. The approach was evaluated by the Support Vector Machines classification.

Another computational method was proposed in [41], referred to as WSMD, in which the optimal set of motifs and the sequences containing them are simultaneously identified through a weakly supervised learning method. Although the method does not incorporate the gapped dependency in the identified motifs, using a similar approach, referred to as LMMO, which maximized the classification accuracy and other relevant metrics (AUC [42]), the algorithm was able to discover a variety of regulatory motifs using a continuous global optimization scheme.

## Materials and methods

### Data sets

We tested the proposed SVM kernel through the binary classification problem, i.e., discriminating a single class of positive (functional) DNA sequences from negative (background) DNA sequences. As the positive signal set, we used the set of 127 CRMs studied in [43], belonging to 114 genes of interest in early *Drosophila* development that exhibit differential expression patterns along the anterio-posterior (AP) axis. The CRM sequences were originally obtained from the data base [44] and extended by 100-bps of flanking sequence in each direction. We generate the negative signal set by retaining the same distribution of sequence lengths and nucleotide compositions as the positive signal set. That is, for each sequence in the positive set, we generate 10 different scrambled versions using random (uniformly-selected) permutations of nucleotides from the original sequence.

Although using a larger negative set generally leads to the problem of an imbalanced data set, we have not observed any imbalance in our data set; as shown in our results, the proposed approach performs with excellent precision-recall curves. If our algorithm resulted in suboptimal performance due to class imbalance on a particular data set, conventional approaches could be applied from the fields of machine learning and bioinformatics, such as data sampling [45] [46], sample weighting [47] [48] [49], cost-sensitive learning [50] [51], kernel-based methods [52], or hybrid approaches [53].

All positive and negative data sets used in this study are available from http://www.columbia.edu/~ae2321/DmelCRMs.zip.

### Identifying top-enriched features

Through SVM training, one can investigate the enrichment of the individual features (gapped *k*-mers) observed in a given input sequence. For this, we use all positive and negative data sets and train the SVM with the proposed kernel. The elements *w*(*n*), *n* = 1, …, *N* in the resulting decision (weight) vector $w=\left[w\left(1\right),\ldots ,w\left(N\right)\right]\in R^{N}$ may indicate the relative discriminative power of the corresponding features, whereby the class of a test example mapped into this feature space $x=\left[x\left(1\right),\ldots ,x\left(N\right)\right]\in R^{N}$ is obtained through the sum of the weighted frequencies of its sequence features, i.e. the sign of the inner product $w^{T}x=\sum_{n=1}^{N}w\left(n\right)x\left(n\right)$. Following this intuition, given the test sequence ***x*** we define the enrichment score of the *n*-th feature in this sequence by *r*(*n*) = *w*(*n*)*x*(*n*) and the score vector corresponding to all features by $r=\left[r\left(1\right),\ldots ,r\left(N\right)\right]\in R^{N}$.

Considering the DNA sequence data, different subsets of features may be enriched in different sequences due to the underlying degeneracy of the binding sequence [54]. In addition, since the nature of DNA binding prefers a “consensus” for particular nucleotides rather than the exact binding sequence, *k*-mers containing a preferred core sequence with certain mismatches may also be enriched [31]. Thereby, it is intuitive to expect that a number of top-enriched features estimated for a given sequence may represent binding sites for the underlying regulatory factor(s) driving the CRM’s primary function [36].

To investigate this, we calculate the enrichment vectors for every positive sequence used in the SVM training (i.e., CRM data set), i.e., {***r***_*i*_, *i* = 1, …, *P*}. For each given sequence, we select the top-enriched features residing above a cutoff weight (0.005) and those belonging to the same feature set (gapped *k*-mer model) are used to construct the relevant “gapped *k*-mer motif”. For example, suppose that in the top-enrichment results there are only two features *ANT* and *TNT* belonging to the set $A_{5}^{3}$, then the corresponding gapped *k*-mer motif of $A_{5}^{3}$ will be (*A*/*T*)*NT*. In fact, such a motif may be the fragment of a real DNA binding motif (i.e., transcription factor binding site), whereby using this *k*-mer motif one can search for the possible hits in a known motif data base. By this motivation, we generated all such motif fragments estimated for each given sequence in the CRM data set.

### Filtering out potential false positive gapped *k*-mers (false discovery-based feature elimination)

**Fig 1:**
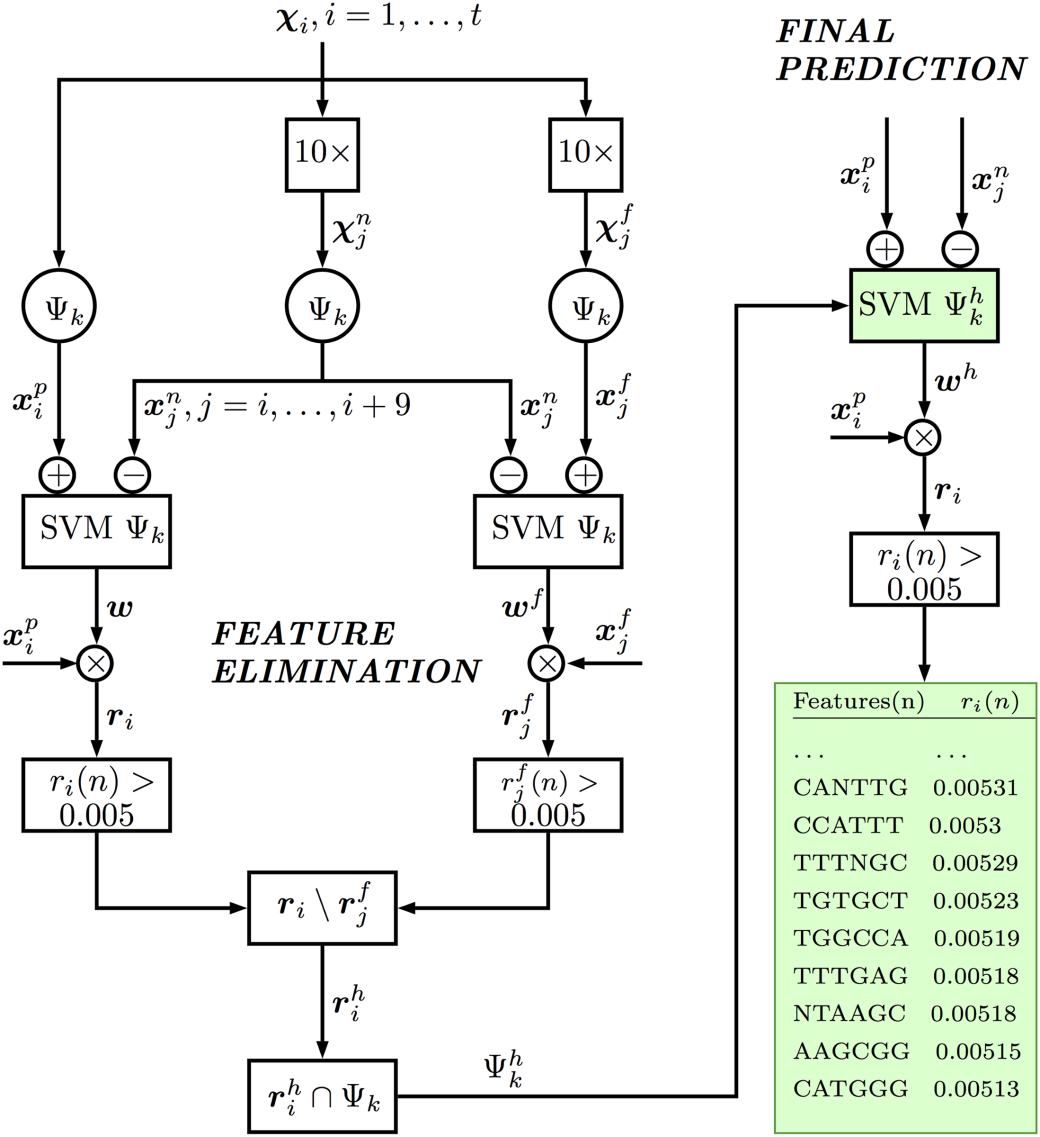
SVM with feature elimination based on the discovery of false enrichments. Feature enrichments (***r***) are estimated by SVM learning and a cutoff threshold. Then the false enrichments are detected and eliminated through using the background (scrambled) positive sequences ($\chi_{j}^{f}$) in the SVM learning. After elimination, the remaining features ($r_{i}^{h}$) are used in the final SVM model $\Psi_{k}^{h}$.

It is known that certain sequence patterns may be found more frequently in the genomic background, entailing the danger, for the above procedure, of falsely discovering such patterns (features) and predicting the subsequent (false) motif hits [55–57]. To address any false discovery caused by the underlying nucleotide composition, we set out to eliminate the features that putatively lead to false enrichments. This is done by replacing the positive inputs with a set of “false” positive sequences to identify an ensemble of corresponding falsely-enriched features (Fig 1). That is, we replace each positive training sequence ***χ***_*i*_, *i* = 1, …, *t* with 10 scrambled versions of it ($\chi_{j}^{f},j=i,\ldots ,i+9$), and use this expanded (false) positive set for training the SVM with the proposed kernel; then we estimate the top-enriched gapped *k*-mers (with $r_{j}^{f}\left(n\right),n=1,\ldots ,N$) for each of the false positive sequences $\chi_{j}^{f},j=1,\ldots ,10t$, and discard those residing below the cutoff weight (0.005), i.e., set $r_{j}^{f}\left(n\right)=0$ if $r_{j}^{f}\left(n\right)<0.005$.

One should note that the 127 CRMs in the positive set are not an exhaustive list of CRMs driving expression of genes in early *Drosophila* development that exhibit differential expression patterns along the AP axis [43, 44]. Therefore, to obtain “false” positive sequences, we randomly scramble the positive sequences, retaining the same number of A, C, G and T nucleotides, opposed to a strategy of choosing random sequences from the genome [11, 12, 58].

### Comparing top-enriched features with those in JASPAR

After filtering, we compared the individual gapped *k*-mers against a relevant motif data base (i.e., JASPAR’s core data base for insects) [59] to elucidate the putative regulatory factors. For this task, we used the motif comparison tool Tomtom [60] which calculates statistical measures (p-value) to quantify the similarity between two motifs. For each sequence, we obtained all the motif hits found by Tomtom and retained those with a significant p-value score < 10^−3^.

To improve the filtering procedure, we further discard “insignificant” false positives by filtering out any gapped *k*-mer which does not lead to a significant JASPAR hit. That is, for each false positive input $\chi_{j}^{f},j=1,\ldots ,10t$, we construct all enriched gapped *k*-mer motifs and search the motif database via Tomtom. If a gapped *k*-mer motif results in a significant hit to a JASPAR motif (*p*-value < 0.001), then we keep this gapped *k*-mer motif, assuming that it consistently appears on the genomic background; otherwise if the motif is not found in JASPAR, we discard that motif, regarding it as an “insignificant” false positive.

The remaining gapped *k*-mers (features) are referred to as “false” and are used to remove the corresponding features found in the positive training sequence’s top-features. That is, we filter out the *n*-th gapped *k*-mer (set *r*_*i*_(*n*) = 0) if it belongs to one of the “gapped models” of the falsely-enriched features (corresponding to $r_{j}^{f}\left(n\right)$) in the respective scrambled copies *j* = *i*, …, *i* + 9. After removal, the remaining gapped *k*-mers (with enrichment scores $r_{i}^{h}\left(n\right)>0.005$) at each input sequence are assumed to be “high-confidence” predictions, whereby we constrained the feature set to this collection, i.e., $\Psi_{k}^{h}=\left(\cup_{i}I\left(r_{i}^{h}\right)\right)\cap \Psi_{k}$ where $I(r_{i}^{h})$ represents the indices of the remaining features with $r_{i}^{h}\left(n\right)>0.005,n\in \left\{1,\ldots ,N\right\}$ (Fig 1).

### Cross-validation

We assess the performance of the proposed folded *k*-spectrum kernel through cross-validation tests. During this procedure, a true positive was defined as a CRM from the original set of 127 ‘positive’ CRMs that was not used in the initial training step but was detected by the algorithm (i.e., correctly classified as a positive sequence). Similarly, a false negative was defined as a CRM from the original set of 127 ‘positive’ CRMs that was not used in the initial training step and was not detected by the algorithm (i.e., incorrectly classified as a negative sequence). Thus, any CRM with a low number of false negatives after this analysis represents a CRM that, even when left out of the training data, can still be detected as a positive sequence.

Since our data set consists of experimentally verified CRMs, we operate under the assumption that the set contains a large number of TFBSs [44]. Therefore, for a valid assessment of performance, we implement a 10-fold cross validation (i.e., we train the algorithm on a large majority of the inputs and test it on the remaining subset).

Given a set of input sequences (i.e., the CRM data set) with positive and negative class labels we randomly divide them into 10 disjoint subsets by retaining (approximately) equal proportions of the positive/negative sequences. At each fold, one different subset is left for testing and the other 9 subsets are used for training the SVM with the proposed folded *k*-spectrum kernel. We note that the false positive features are eliminated from the feature set and the feature map is constrained to $\Psi_{k}^{h}$ following the procedure described in the previous sections.

**Fig 2:**
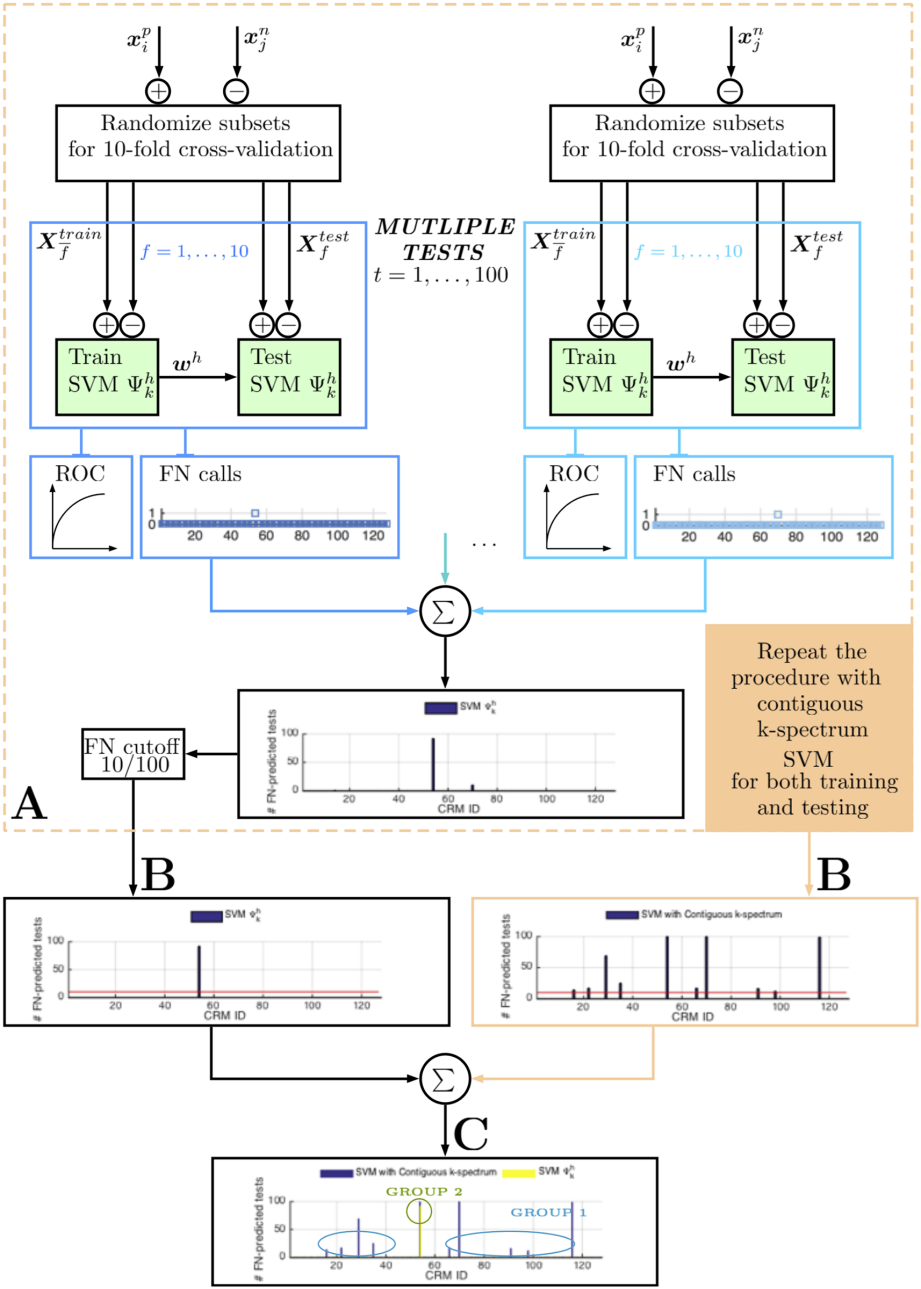
Discovery of the different groups of sequences in the two methods’ (contiguous *k*-mer SVM, folded *k*-mer SVM) predictions, based on the differential FN (false negative) calls cumulated over multiple cross-validation tests. For each method, multiple randomized leave-sets-out cross-validation tests are performed and an ensemble of FN-predicted sequences are determined (**A**). The sequences that are called negative above 10% call rate (FN cutoff) are then reported (**B**). The reported sequences of both methods are compared and the intersecting/differing groups of sequences are determined (**C**).

After processing 10 folds, each input sequence is tested (classified) once. We then obtain those class predictions and determine the true/false predictions for the positive and negative sequences. The corresponding ROC curve and Area Under Curve (*AUC*) score are then generated (Fig 2).

We re-evaluate these predictions in the repeated experiments in order to average out the influence of randomization on the test subsets. We repeat the above procedure 100 times by randomly dividing the data in each experiment, then check the cumulative “false negative” prediction of each input sequence, which represents the number of times a trained classifier fails to detect that sequence accurately. This evaluation elucidates the predictive power of the “gapped *k*-mer” features on the individual input sequences.

We want to identify the group of CRMs in which the classifier performs with low accuracy, i.e., through repeated experiments the classifier “consistently” fails to detect those CRMs and results in a high number of false negative (FN) calls. The consistency can be defined by setting a threshold in the FN call rate, i.e., FN>10/100. For example, if a classifier (falsely) predicts a positive test sequence as negative in at most 10% of the repeated (randomized) cross-validation experiments, we can assume that the classifier is able to predict that sequence with high (90%) accuracy. Fig 2 displays the (sequence-specific) prediction accuracy of the classifiers, the contiguous *k*-spectrum kernel and the folded *k*-spectrum kernel, based on the 10% FN call rate cutoff. Subsequently, one can determine the groups of CRMs that the methods (contiguous *k*-mer vs. folded *k*-mer) perform differently or similarly, and investigate the underlying result of each classifier on these CRM groups.

## Results

### Identification of enriched motifs

We implemented the folded *k*-spectrum kernel approach using a set of 127 CRMs responsible for the regulation of 114 genes exhibiting differential expression during early *Drosophila* development. These CRMs are referred to as our ‘positive sequences’, as each of them has been experimentally shown to regulate gene expression along the AP axis, and thus each is thought to contain motif(s) corresponding to TFBSs present in the early embryo [61]. Those referred to as ‘negative sequences’ during our SVM training procedure have been constructed by randomly scrambling the positive sequences while retaining each sequence’s original nucleotide distribution (see Methods for more details).

Based on the cross-validation tests described in Fig 2 (with 10% FN call rate cutoff), our algorithm resulted in the identification of three very interesting subsets (groups) of these CRMs:

Group 1 those that are correctly detected as belonging to the ‘positive sequence set’ by gapped *k*-mers (i.e., folded *k*-spectrum kernel), but not detected by contiguous *k*-mers (i.e., contiguous *k*-spectrum kernel),
Group 2 those that are incorrectly identified as belonging to the ‘negative sequence set’ by both gapped *k*-mers and contiguous *k*-mers, and
Group 3 those that are correctly detected as belonging to the ‘positive sequence set’ by both contiguous *k*-mers and gapped *k*-mers.

**Fig 3:**
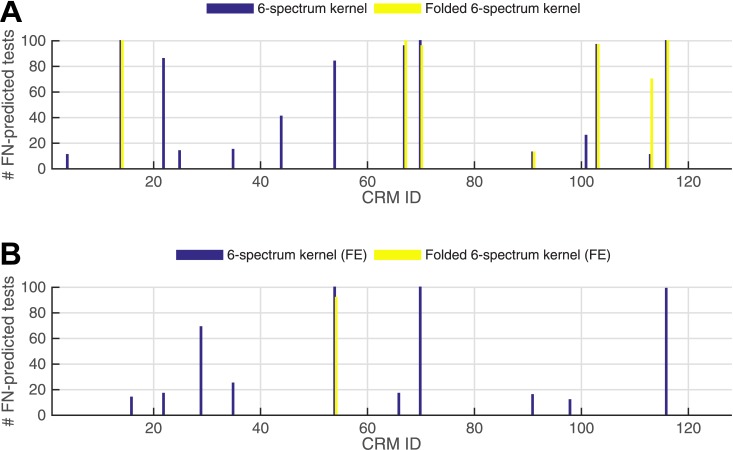
Classification performance of the (contiguous) *k*-spectrum kernel vs. folded *k*-spectrum kernel without (A) and with (B) feature elimination (FE) procedure. For each method, 100 randomized leave-sets-out cross-validation tests are performed and an ensemble of FN-predicted sequences are determined. The bar plot displays the total number of times a given (positive) CRM sequence is classified as negative.

We observed that 7 CRMs fall into Group 1, 7 fall into Group 2, and the remaining 113 fall into Group 3 (Fig 3A, S1 Table). One should note that none of the 127 CRMs were correctly detected by contiguous *k*-mers but not by gapped *k*-mers due to the fact that contiguous *k*-mers are included when considering all possible gapped *k*-mers. The subset of CRMs contained in Group 1 may contain crucially important TFBSs with gapped motifs, which are undetectable using the standard approaches. Therefore, we focus our attention on those motifs (gapped *k*-mers) enriched in the Group 1 CRMs (S2 Table), and include the enriched gapped *k*-mers in the Group 2 and 3 CRMs in the Supplementary Information (S3 Table).

The results in S2 Table give a comprehensive picture of all of the gapped *k*-mers found to be enriched (weight > 0.005) in the Group 1 CRMs. However, to further investigate the importance of particular gapped *k*-mer models, we combined these results into S4 Table, which gives each gapped *k*-mer model found to be enriched, the number of enriched gapped *k*-mers that fell into that gapped *k*-mer model, and the cumulative weight of those enriched gapped *k*-mers. Note that the most highly enriched gapped *k*-mers in each of the Group 1 CRMs are those containing only a small number of gaps (i.e. most have a single nucleotide gap), suggesting that these gaps are contributing significantly to TFBS identification.

### Removing false positives

There are some sequence features that may be found to be more abundant than others, even in the genomic background sequence, due to the sequence composition, although they do not represent TFBSs. Thus, in an attempt to remove these ‘false positive’ motifs (spurious gapped *k*-mers), we have used a filtering procedure (see Methods for more details). This procedure has eliminated approximately 88% of the total gapped *k*-mer features that were previously found to be enriched. S5 and S6 Tables, and all remaining figures correspond to these filtered results.

After filtering, 9 CRMs fall into Group 1, 1 falls into Group 2, and the remaining set of 117 CRMs fall into Group 3 (Fig 3B, S7 Table). Note that 3 CRMs moved from Group 2 to Group 1 after filtering (i.e., the filtering procedure allowed them to be correctly detected as belonging to the ‘positive sequence set’ by gapped *k*-mers, but still incorrectly identified them by contiguous *k*-mers), only 1 CRM moved from Group 1 to Group 2 after filtering (i.e., the filtering procedure caused the gapped *k*-mers to lose their ability to correctly identify the CRM). Thus, overall the filtering procedure improves the performance of the gapped *k*-mers when compared to the contiguous *k*-mers.

S5 Table shows the gapped *k*-mers found to be enriched in the Group 1 CRMs using the 127 positive CRM sequences for training after eliminating features characterized as ‘false’ during this procedure.

The overall number of enriched gapped *k*-mers in the Group 1 CRMs decreases after filtering (from an average of approximately 176 enriched gapped *k*-mers per CRM to 90 enriched gapped *k*-mers per CRMs, S2 vs. S5 Tables), but there still remain a large number of enriched gapped *k*-mers.

Again, to investigate the importance of particular gapped *k*-mer models, we combined the filtered results into S6 Table, the number of enriched gapped *k*-mers within that model, and the cumulative weight of those enriched gapped *k*-mers. An interesting observation is that those found to be highly enriched after filtering have a small number of gaps, i.e., *αααNαα*, *αNαααα*, *ααααNα*, *ααNααα* etc., where *α* ∈ {*A*, *C*, *G*, *T*}.

However, one should note that the gapped *k*-mers that belong to the contiguous model *αααααα* were characterized as false enrichments in all cases.

### Improved performance of the folded *k*-spectrum approach

To validate our model and compare it to the traditional *k*-spectrum kernel, we conducted a set of cross-validation experiments. This was done by determining the number of true positive/ false negative class predictions of sequences through a random leave-sets-out analysis (see Methods for more details).

After repeating the leave-sets-out analysis 100 times using both the traditional *k*-spectrum kernel as well as the folded *k*-spectrum kernel (with feature elimination) during the initial training, leaving out subsets of the positive as well as negative (scrambled) CRMs for testing, the resulting number of false negatives found are shown in Fig 3B and in S7 Table. One should note that the SVM with folded *k*-spectrum performs better than the traditional SVM with *k*-spectrum method in all CRMs.

**Fig 4:**
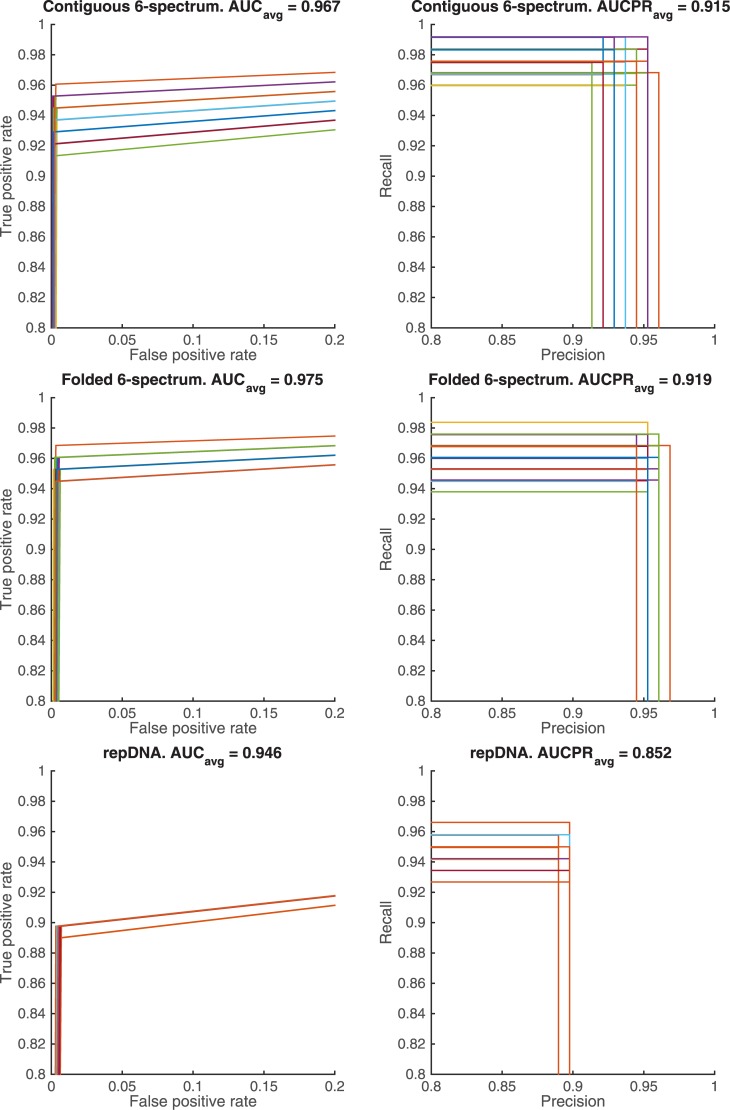
Superimposed performance curves, ROC (left) and PR (right), of the 100 cross-validation tests by SVM learning with contiguous *k*-spectrum kernel (top), folded *k*-spectrum kernel (middle), and repDNA features (lower). Note that many of the ROC curves overlap due to the very small number of false positives found. Different colors represent different cross-validation results.

**Fig 5:**
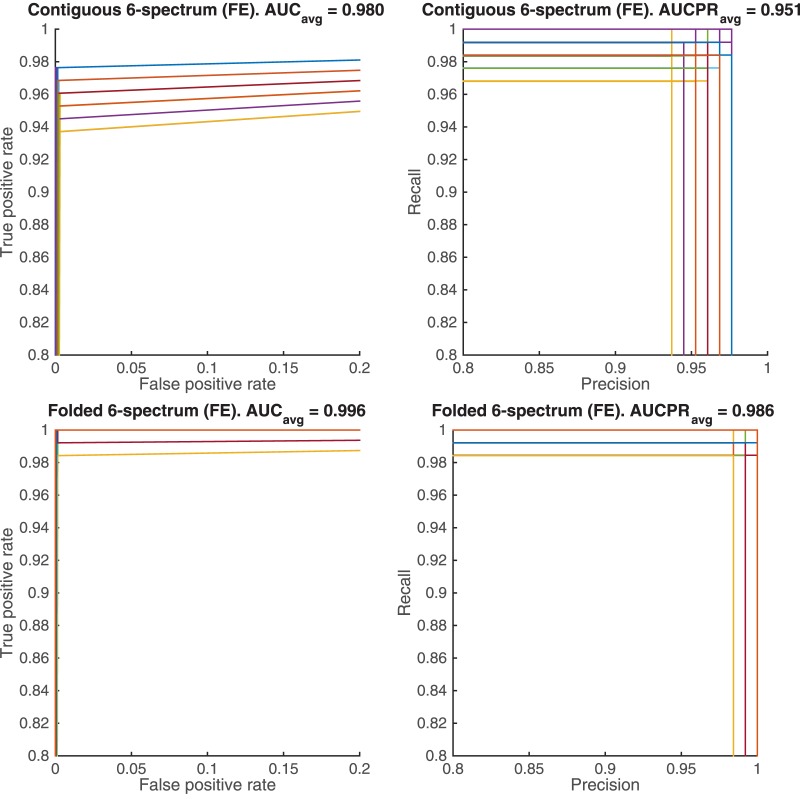
Superimposed performance curves, ROC (left) and PR (right), of the 100 cross-validation tests by using the feature elimination (FE) in SVM learning with contiguous *k*-spectrum kernel (above) vs. folded *k*-spectrum kernel (below). Note that many of the ROC curves overlap due to the very small number of false positives found. Different colors represent different cross-validation results.

The overall performance of the classifiers are evaluated through the ROC curves, i.e., false positive rate $\left(\frac{FP}{FP+TN}\right)$ vs. true positive rate $\left(\frac{TP}{TP+FN}\right)$ and the precision-recall curves (PR), i.e., precision (or positive predictive value) $\left(\frac{TP}{TP+FP}\right)$ vs. recall (or true positive rate). Fig 4 shows the superimposed ROC/PR curves of the 100 binary classification results, with the corresponding average Area Under the Curve scores for ROC (*AUC*_*avg*_) and for PR (*AUCPR*_*avg*_). Fig 5 shows the results corresponding to the top four panels of Fig 4 when the feature elimination procedure is applied. The folded *k*-spectrum approach clearly outperforms the contiguous *k*-spectrum in terms of *AUC* and *AUCPR* scores, with the feature elimination procedure further improving the classifier’s performance.

We also compared our results to those of the repDNA algorithm [35] by generating the *k*-mers (with *k* = 1, …, 6) and a set of features incorporating the gapped dependency, i.e., dinucleotide-based autocovariance (DAC) features, dinucleotide-based cross covariance (DCC) features, and pseudo dinucleotide composition (PseDNC) features. We applied 10-fold cross-validation using the binary classifier SVM, and repeated this test 100 times. The results illustrate that the folded *k*-spectrum approach also outperforms the repDNA algorithm in terms of both *AUC* and *AUCPR* scores (Fig 4).

### Enriched motifs correspond to known TFBSs

For the enriched motifs found after filtering in the 9 Group 1 CRMs, we set out to gain some insight into what particular TFs these motifs may be binding *in vivo*. The improved predictive power obtained by the gapped *k*-mer models in those CRMs suggests the presence of TFs with strong nucleotide interdependencies in their binding sites. To identify these candidate TFs, we analyzed the enriched gapped *k*-mers using the insect motif database, JASPAR, along with the widely used comparison tool, Tomtom, quantifying the similarity between our enriched motifs (gapped *k*-mer sequences) and known motifs for regulatory factors [59, 60].

We analyzed all 817 enriched gapped *k*-mers found in the Group 1 CRMs, and found 136 significant hits using JASPAR (*p*-value < 10^−3^, see S8 Table). The most prominent feature of this analysis was that a large number of the enriched gapped *k*-mers correspond to TFBSs for TFs known to regulate genes involved in early AP patterning in *Drosophila*.

#### TFs known to regulate AP patterning genes

The hits found by all gapped *k*-mers correspond to 29 different TFs, 14 of which are known to be involved in regulating AP patterning genes [2, 62]. These include BCD, BTD, GSC, OC, KR, TTK, KNI, TLL, HKB, H, SLP, OPA, ODD, and RUN. It is not surprising to find such a large number of the significant hits corresponding to TFs known to regulate AP patterning genes since the 127 CRMs used for the analysis are known to regulate genes that are differentially expressed along the AP-axis. However, it is worth noting that through this analysis some interesting gapped dependency patterns emerged. As an example, we focus on BCD, the TF found with the second highest number of occurrences in Group 1 CRMs.

**Fig 6:**
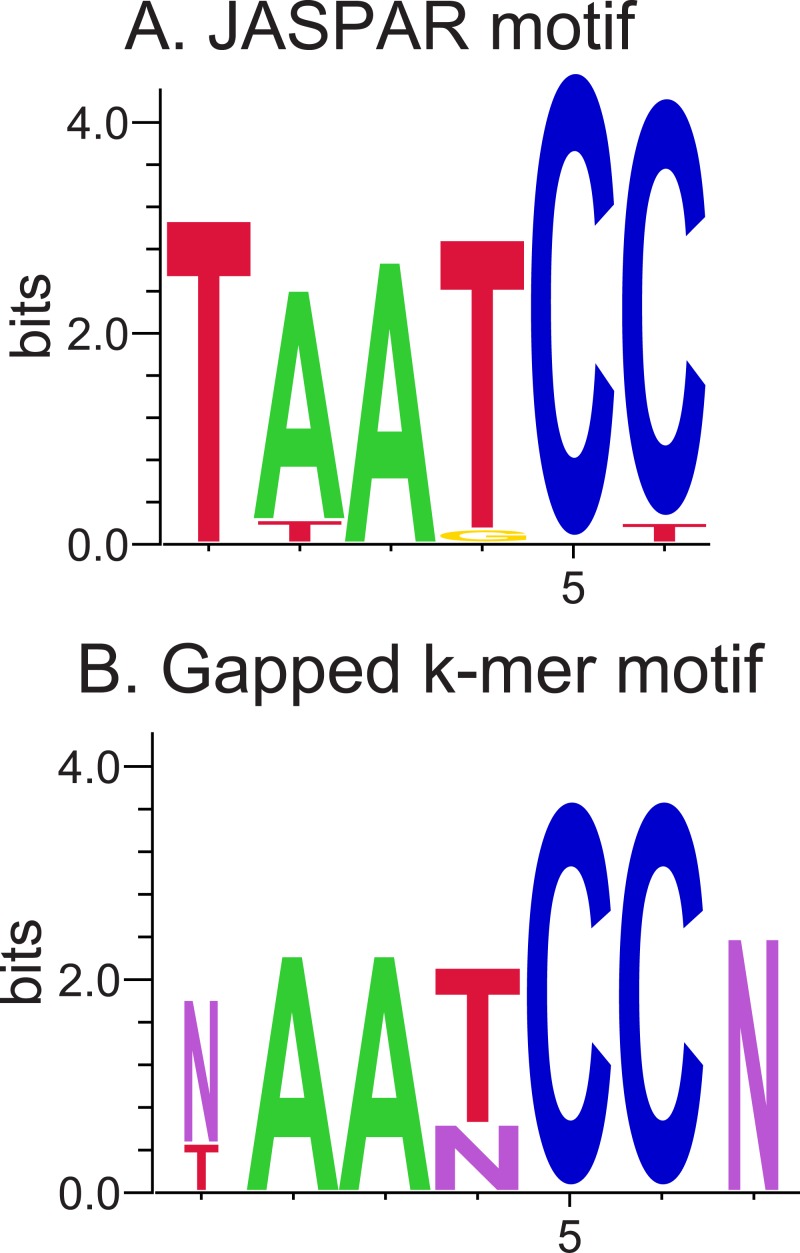
Weblogos of the JASPAR motif (A) and the enriched gapped *k*-mer motifs (B) corresponding to the BCD TFBSs. Note that in the weblogos, A, C, G, and T represent the corresponding nucleotides, while purple N’s in (B) represent gaps introduced by the enriched gapped *k*-mers.

The enriched gapped *k*-mers that were found to be significant with the known BCD motif using Tomtom were aligned and used to construct the weblogo in Fig 6B. One should note that this weblogo is very similar to JASPAR’s BCD motif (Fig 6A), in which the gaps significantly align to the 4th base and the (flanking) 7th base of the JASPAR motif. Although the majority (7) of these aligned enriched gapped *k*-mers have the contiguous 5-mer model (*Nααααα*), the remainder of them (3) have a consistent appearance of the gap in the 4th base, suggesting a non-adjacent (gapped) dependency between nucleotides 2 and 3, and 5 and 6.

One possible explanation for this gapped dependency may be the three-dimensional shape of the DNA when bound by BCD. BCD is a homeodomain TF, and thus is thought to mediate DNA binding through contact primarily with nucleotides in the major groove using the homeodomain recognition helix, although there is evidence of additional DNA contact in the minor groove via the relatively unstructured N-terminal domain [63–65]. Therefore, nucleotides in the transition between the major and minor groove may not be as important when binding. When the BCD binding sites in the JASPAR database, originally identified using bacterial-1 hybrid experiments [62], were analyzed using the newly developed and validated high-throughput approach, DNAshape, [66] they were predicted to contain a significantly smaller minor groove width estimated on the 4th base [67]. We believe the gap we observe in the 4th base (and the entailing dependency between the surrounding nucleotides) may be due to this reduced minor groove width, signifying the nucleotide position where the transition occurs from the major to minor groove.

## Discussion

Approaches to binding site prediction from DNA sequence data have been developed, and improved upon for decades. The development and implementation of the novel approach laid out in this manuscript has led us to some very interesting conclusions. Most striking, and probably most important to the general bioinformatic community, is the illustrated advantages that this new approach, which incorporates gapped dependency, has shown over existing SVM approaches. To highlight the success of this approach, note that 126 of the 127 CRMs tested were correctly identified as belonging to the ‘positive sequence set’ by this new algorithm, while 9 of those were not correctly identified by the previous contiguous *k*-mer approach, and the only remaining CRM was incorrectly identified by both approaches. Thus, our new algorithm outperformed the previous algorithm over the complete set of 127 CRMs (Fig 3B). We also found, through a rigorous set of cross-validation tests, that our novel approach outperformed the previous approach in terms of the *AUC* measurements (average of 0.996 vs. 0.98, Fig 5), as well as in terms of the *AUCPR* measurements (average of 0.986 vs. 0.951, Fig 5).

Beyond showing the advantage this approach holds over previous SVM approaches, we have also illustrated its utility on the specific set of CRMs tested, validating the method and raising new biological hypotheses regarding the nature of DNA-protein binding. The 127 CRMs chosen for this study belong to 114 genes which exhibit differential expression patterns along the AP-axis in early *Drosophila* embryos. When comparing the gapped *k*-mers found to be enriched using our algorithm to those in the insect motif database, JASPAR, we found that 14 of the 29 TFs found were indeed known to be involved in regulating AP patterning genes. Although not all TFs found were known to be involved in the regulation of AP patterning genes, the appearance of many of these other TFs can still be explained in a reasonable biological context. For example, BRK and MAD are thought to compete for binding sites effecting Dpp signaling events in early *Drosophila* development, and our results support this as we have found them enriched on the same enhancers. We have also found enrichment of CTCF binding site sequences within these 127 known CRMs, which has been shown to be involved in enhancer-promoter looping. These results all lead us to believe that the motifs we are identifying, in many cases, likely represent true TF binding sites.

## Conclusion

This study has introduced a novel approach to motif identification, which builds upon previous approaches in machine learning to allow for a less biased approach to binding site discovery. We have shown its ability to predict TF binding sites on a set of 127 CRMs, and have offered some insight into the molecular basis for its success. In the future, it will be very interesting to see similar analyses performed on various other sets of sequences, possibly including sequences from different species and different time points in development. Such studies could help in answering a deeper biological question of whether universal rules exist governing binding site preference, strength, and flexibility.

## Supporting information

S1 Table Classification performance of the (contiguous) k-spectrum kernel vs. folded k-spectrum kernel (Fig 3). (XLS)

S2 Table Gapped k-mers enriched in the Group 1 CRMs. (XLS)

S3 Table Gapped k-mers enriched in each of the 127 CRMs. (XLS)

S4 Table The gapped k-mer models enriched in the Group 1 CRMs with total counts and cumulative weights of the corresponding motifs from S2 Table. (XLS)

S5 Table The gapped k-mers enriched in the Group 1 CRMs by using the feature elimination procedure in the SVM methods. (XLS)

S6 Table The gapped k-mer models enriched in the Group 1 CRMs by using the feature elimination procedure in the SVM methods. (XLS)

S7 Table Classification performance of the (contiguous) k-spectrum kernel vs. folded k-spectrum kernel with feature elimination (FE) procedure (Fig 4). (XLS)

S8 Table Motif comparison results with the enriched gapped k-mers in the Group 1 CRMs (from S5 Table). (XLS)

S1 File Folded k-spectrum program. Matlab implementations of the algorithms described in the Methods and Results sections.
(ZIP)
