# Supplementary material for: The folded k-spectrum kernel: A machine learning approach to detecting transcription factor binding sites with gapped nucleotide dependencies
Source: PLoS One. 2017 Oct 5;12(10):e0185570. doi: 10.1371/journal.pone.0185570 (PMC5628859; doi:10.1371/journal.pone.0185570)
Supplement: S1 File — Matlab implementations of the algorithms described in the Methods and Results sections. (ZIP) [file pone.0185570.s009.zip › S1_File/Readme.pdf]

THE FOLDED K-SPECTRUM KERNEL

SOFTWARE INSTRUCTIONS

Main program

The main program is implemented in Matlab, *pipelineSVMFE.m*. For a given set of input sequences it executes the proposed SVM method (Figure 1) in the accompanying paper. The program mainly depends on four Matlab functions which are self-contained and documented, *mappingFunction.m*, *mappingFoldedKspec.m*, *mappingKspec.m*, and *binarySpace.m*. It also depends on the *Tomtom* program where the instructions can be found at `http://meme-suite.org/tools/tomtom`. The executable program *tomtom* should be placed within the same directory.

Inputs:

- **positive_sequences**, a set of DNA sequences (χ_i) given in fasta format
- **r_cutoff**, a threshold value to filter out weak feature enrichments, 0.005 (default)

Outputs:

- **features_final**, a set of enriched gapped k-mer features (Figure 1)
- **ri_final**, the corresponding enrichment scores, r_i

Feature Elimination

For the elimination phase ($r_i \setminus r_j^f$) we considered two options.

Basic: eliminate any feature n with $r_i(n) > 0$, if the corresponding $r_j^f(n) > 0$, $j = i, \ldots, i+9$.

Advanced (default): eliminate any feature n with $r_i(n) > 0$, if it belongs to the "gapped model" of any z with $r_j^f(z) > 0$.

In the *Advanced* option, before the elimination takes place, we run Tomtom for each false enrichment z and retain it if Tomtom finds significant similarity with a particular JASPAR motif ($pval < 0.001$), otherwise we set $r_j^f(z) = 0$, considering them as noise rather than background sequence patterns.

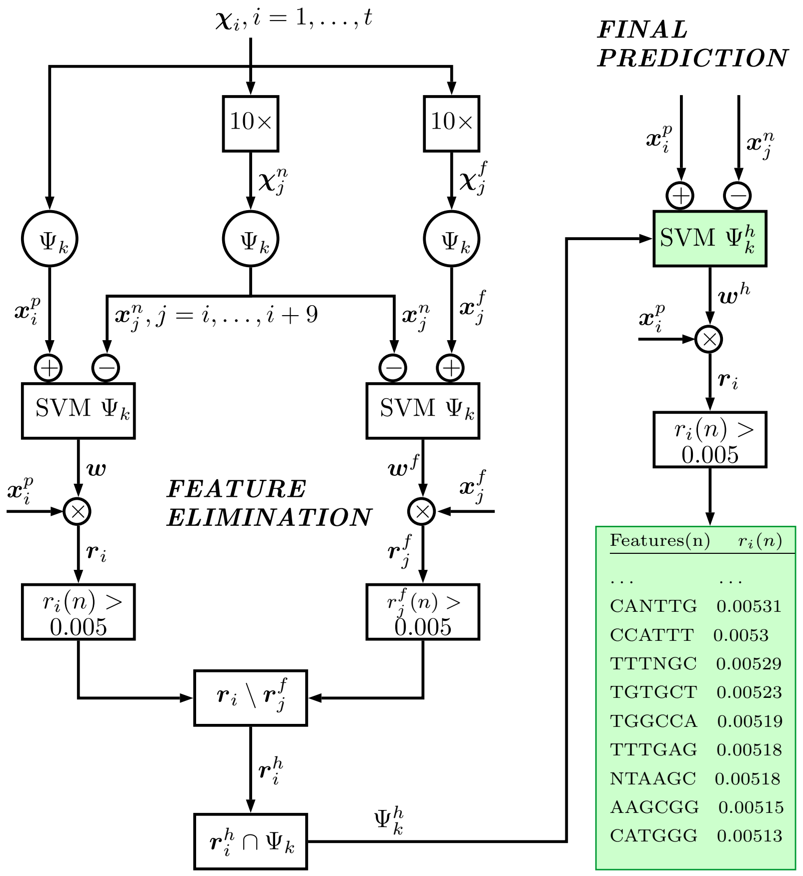

FIGURE 1. SVM with feature elimination based on the discovery of false enrichments
